# Discovery of p-Coumaric Acid as a Candidate Cholesterol-Lowering Factor in Germinated Brown Rice via Untargeted Metabolomics Combined with a Cholesterol-Induced HepG2 Cell Model

**DOI:** 10.3390/foods15081344

**Published:** 2026-04-13

**Authors:** Ningxin Ding, Xiangyu Pang, Yixuan Yan, Mingcong Fan, Haifeng Qian, Li Wang, Yan Li

**Affiliations:** State Key Laboratory of Food Science and Resources, National Engineering Research Center for Functional Food, School of Food Science and Technology, Jiangnan University, 1800 Lihu Avenue, Wuxi 214122, China; 6230111021@stu.jiangnan.edu.cn (N.D.); pxyu000909@163.com (X.P.); 7240112101@stu.jiangnan.edu.cn (Y.Y.); 8202009403@jiangnan.edu.cn (M.F.); qianhaifeng@jiangnan.edu.cn (H.Q.)

**Keywords:** germinated brown rice, cholesterol metabolism, metabolomics, p-coumaric acid

## Abstract

Hypercholesterolemia is a major modifiable risk factor for cardiovascular disease. Although germinated brown rice (GBR) is rich in bioactive constituents and has shown preliminary lipid-regulating potential, the active compounds and underlying mechanisms remain unclear. Therefore, this study comparatively investigated extracts from four GBR cultivars and evaluated their cholesterol-regulating activity using untargeted metabolomics in combination with a cholesterol-induced HepG2 cell model. After 36 h of germination, the extracts showed significantly increased total phenolic content, total flavonoid content, and antioxidant activities (*p* < 0.05), with cultivar Q showing the strongest response. In the in vitro cholesterol-induced HepG2 cell model, GBR extract at 150 μg/mL significantly reduced intracellular total cholesterol (TC), triglyceride (TG), and low-density lipoprotein cholesterol (LDL-C) levels by approximately 69%, 73%, and 46%, respectively, while increasing high-density lipoprotein cholesterol (HDL-C) levels by approximately 125% (all *p* < 0.05). Untargeted metabolomics and subsequent validation suggested that p-coumaric acid is a candidate bioactive compound contributing to the cholesterol-lowering effect of GBR. Further analysis indicated that this effect may be associated with modulation of the mRNA expression levels of SREBP2, HMGCR, and LDLR. These findings indicate that germination enhances the bioactivity of brown rice in a cultivar-dependent manner and provides supporting evidence for the potential application of GBR as a functional food ingredient for lipid management.

## 1. Introduction

Cholesterol serves critical physiological functions, including acting as a fundamental structural component of cell membranes and as a precursor for the synthesis of vitamin D, steroid hormones, and bile acids [1]. In contrast, extensive research has established that hypercholesterolemia, characterized by elevated plasma concentrations of TC and LDL-C, is a major risk factor for atherosclerosis and subsequent cardiovascular diseases [2]. Currently, statins are the primary pharmacological therapy for lowering cholesterol levels. However, their long-term use may be associated with adverse effects such as myalgia and hepatotoxicity. Evidence indicates that dietary consumption of whole grains is associated with a reduced risk of mortality from cardiovascular diseases [3], while also providing protective effects against various chronic conditions [4].

Rice (*Oryza sativa* L.) is a major cereal crop worldwide and a staple food in Asia. Brown rice (BR), one of the most common whole grains, has attracted considerable attention in recent years because of its nutritional value and processing potential. Compared with traditional polished white rice, BR retains the germ and bran layers [5]. Previous studies have shown that increased BR consumption may help prevent and ameliorate various diseases, including cardiovascular diseases, hyperlipidemia, hypertension, diabetes, and obesity. However, its relatively coarse texture and lower palatability compared with polished white rice have limited its widespread consumer acceptance. These limitations have prompted researchers to explore effective processing strategies. Existing studies have demonstrated that the sensory quality of BR can be improved through physical processing methods, such as parboiling, microwave treatment, and extrusion puffing; biological treatments, such as enzymatic hydrolysis, fermentation, and germination; as well as emerging technologies, including static magnetic fields and high hydrostatic pressure [6]. Among these techniques, germination stands out because of its operational simplicity and cost-effectiveness. Its ability to simultaneously improve sensory quality and enhance nutritional value renders it a highly promising processing strategy.

Previous studies have demonstrated that germination activates the endogenous enzyme system in BR, resulting in a 40–60% increase in total phenolic content and a 3–5-fold increase in γ-aminobutyric acid (GABA) content. In addition, appropriately increasing the soaking temperature before germination can enhance phytase activity and accelerate the degradation of phytic acid, thereby improving the mineral bioavailability in BR. These findings suggest that germination is an effective strategy for enhancing the nutritional quality of BR [7].

In recent years, numerous studies have demonstrated the cholesterol-lowering potential of GBR. Hao et al. [8] found that pre-germinated brown rice extract (PGBRE) improved dyslipidemia in mice fed a high-fat diet by reducing TG, LDL-C and non-HDL cholesterol levels, increasing HDL-C levels, and lowering the risk of atherosclerosis. The underlying mechanisms may involve inhibiting the expression of 3-hydroxy-3-methylglutaryl-CoA reductase (HMGCR), sterol regulatory element-binding protein 1 (SREBP-1), stearoyl-CoA desaturase-1 (SCD-1) and fatty acid synthase (FAS) in the liver, thereby reducing hepatic cholesterol and lipid synthesis; upregulation of low-density lipoprotein receptor (LDLR) and cholesterol 7α-hydroxylase (CYP7A1), which promotes cholesterol metabolism; and reduction in lipid absorption while increasing the fecal excretion of TG, TC and bile acids. In addition, some bioactive constituents in BR, such as ferulic acid, GABA, and γ-oryzanol, have been reported to be associated with lipid-lowering effects or improvements in cholesterol metabolism. However, most previous studies have focused on a single cultivar or mixed samples, without adequately considering the metabolic differences among rice cultivars. In fact, existing studies have demonstrated clear differences in the dynamic changes in metabolites during the germination of BR from different genotypes [9]. Such cultivar-dependent variation, together with the compositional changes induced by germination, is likely to lead to differences in the final biological activity. Therefore, comparative studies across different cultivars are of great significance for screening high-quality functional raw materials.

Metabolomics has emerged as a powerful analytical platform for characterizing bioactive constituents in complex food matrices. High-throughput techniques, particularly UPLC-QTOF-MS, enable the simultaneous detection and quantification of a wide range of metabolites. When combined with multivariate statistical methods such as OPLS-DA, metabolomics can effectively identify key biomarkers [10]. This technology not only enables comprehensive profiling of plant metabolites but also plays an important role in the investigation of secondary metabolites. Furthermore, when integrated with bioassays, it can efficiently screen and identify candidate active compounds with potential functional properties [10]. Accordingly, unlike previous studies that directly assumed a single component to be the dominant active substance, the present study aimed to prioritize candidate bioactive compounds associated with cholesterol-lowering activity in germinated brown rice through a multi-cultivar comparative strategy integrating metabolomics, correlation analysis, and subsequent functional validation.

The four brown rice cultivars used in this study were freshly harvested from field-grown materials provided by Beijing Golden Silver Hua Agricultural Technology Co., Ltd., Beijing, China. All four cultivars belong to the indica rice type, which is one of the major rice types widely cultivated and consumed in southern China. Therefore, these cultivars were selected as representative materials for comparing cultivar-dependent differences in phenolic accumulation, antioxidant activity, and cholesterol-lowering potential during germination. Against this background, this study employed a multi-cultivar comparative metabolomics strategy combined with molecular and cell biology techniques to further investigate the regulatory effects of GBR and its underlying mechanisms.

## 2. Materials and Methods

### 2.1. Material and Chemicals

In this study, BR from four widely cultivated non-aromatic rice cultivars in China provided by Beijing Golden Silver Hua Agricultural Technology Co., Ltd. (Beijing, China), were used: Quanyou 607, Jeliangyou 623, Weiliangyou, and Quanyue Yuennong Simiao, hereinafter referred to as Q, J, W and S, respectively. Folin–Ciocalteu reagent, 2,2-diphenyl-1-picrylhydrazyl radical (DPPH), and 2,2′-azino-bis (3-ethylbenzothiazoline-6-sulfonic acid) diammonium salt (ABTS) were purchased from Shanghai Yuanye Bio-Technology Co., Ltd. (Shanghai, China), 25-hydroxycholesterol (purity: ≥98%) was purchased from Shanghai Aladdin Biochemical Technology Co., Ltd. (Shanghai, China) and cholesterol (purity: ≥99%) was purchased from Sigma-Aldrich (St. Louis, MO, USA). The experimental water was ultrapure water, prepared by the Milli-Q Plus water purification system (Milli-Q Plus, Millipore, Milford, MA, USA). All other reagents and chemicals used were of analytical grade.

### 2.2. Preparation of the Germinated Brown Rice

The method by Wu et al. [11] was adopted with slight modifications. BR grains were surface-disinfected with sodium hypochlorite, soaked in distilled water at 30 °C for 12 h (with the soaking water replaced every 4 h), and germinated in a dark incubator at 30 °C and 90% relative humidity for 12, 24, and 36 h. During germination, the grains were placed on four layers of water-soaked gauze, and distilled water was sprayed every 4 h to maintain adequate moisture. The GBR grains were then washed, dried at 50 °C, milled, sieved through a 60-mesh screen, and freeze-dried.

The germination rate was determined at 12, 24, and 36 h of germination. A grain was considered germinated when the radicle protruded through the seed coat by at least 1 mm. At each time point, 100 BR grains were examined, and the number of GBR grains was recorded. The germination rate was calculated according to Equation (1):

(1) $$Germination\;rate(\%)=\frac{Number\;of\;germinated\;grains}{Total\;number\;of\;grains}\times 100$$

For methanol extraction, the powder was mixed with 80% methanol at a ratio of 1:30 (*w*/*v*), shaken at 37 °C for 30 min, and then ultrasonically extracted at 40 °C for another 30 min at a frequency of 40 kHz and a power of 300 W. After centrifugation, the supernatant was collected. The extraction was repeated twice, and the combined supernatants were concentrated by rotary evaporation at ≤45 °C, freeze-dried, and stored at −20 °C.

### 2.3. Determination of Phenolic and Flavonoid Contents and Antioxidant Activity

#### 2.3.1. Determination of Total Phenol Content (TPC)

TPC was determined using the Folin–Ciocalteu method described by Chang et al. [12], with slight modifications. A 200 μL aliquot of the sample was mixed with an equal volume of Folin–Ciocalteu reagent and 800 μL of deionized water. The mixture was incubated in the dark for 6 min, after which 2 mL of 7% Na_2_CO_3_ and 1.8 mL of deionized water were added. After incubation for 90 min, the absorbance of the mixture was measured at 760 nm. TPC was expressed as mg gallic acid equivalents (GAE)/100 g DW.

#### 2.3.2. Determination of Total Flavonoid Content (TFC)

TFC was determined using the colorimetric method described by Chang et al. [12], with slight modifications. A 0.5 mL aliquot of the extract was mixed with 2 mL of deionized water and 0.15 mL of 5% NaNO_2_. After 6 min, 0.15 mL of 10% AlCl_3_ solution was added, and the mixture was allowed to stand for another 6 min. Then, 2 mL of 1 M NaOH was added, and distilled water was added to bring the final volume to 5 mL. After standing for 10 min, the absorbance was measured at 510 nm. TFC was expressed as mg rutin equivalents (RE)/100 g DW.

#### 2.3.3. DPPH Free Radical Scavenging Activity

The DPPH free radical scavenging activity of the extract was determined by the method described by Chang et al. [12], with slight modifications. A 50 μL aliquot of the extract was mixed with 750 μL of DPPH solution (0.076 mM), and the mixture was incubated at room temperature in the dark for 30 min. Subsequently, the absorbance of the mixture was measured at 517 nm. The antioxidant activity was calculated according to Equation (2):

(2) $$\mathrm{DPPH}\;\mathrm{radical}\;\mathrm{scavenging}\;\mathrm{activity}\;\left(\%\right)=\left[\frac{\left(A_{0}-A_{s}\right)}{A_{0}}\right]\times 100$$

where A_0_ is the absorbance of the blank and A_s_ is the absorbance of the sample.

#### 2.3.4. ABTS Free Radical Scavenging Activity

The ABTS free radical scavenging activity of the extract was determined by the method described by Wang et al. [13], with slight modifications. The ABTS^+^ solution was prepared by mixing 7.0 mM ABTS and 4.95 mM potassium persulfate in equal volumes to obtain a stock solution, which was then stored in the dark at 4 °C for 12–16 h. The stock solution was subsequently diluted, and its absorbance was adjusted to 0.70 ± 0.02 at 734 nm. Then, 10 μL of extract was added to 190 μL of ABTS^+^ solution, and the mixture was incubated at room temperature in the dark for 10 min. The absorbance was then recorded at 734 nm. The antioxidant capacity determined by the ABTS assay was calculated according to Equation (3):

(3) $$ABTS\;\mathrm{radical}\;\mathrm{scavenging}\;\mathrm{activity}\;\left(\%\right)=\left[\frac{\left(A_{0}-A_{s}\right)}{A_{0}}\right]\times 100$$

where A_0_ is the absorbance of the blank and A_s_ is the absorbance of the sample.

### 2.4. Scanning Electron Microscopy (SEM)

Scanning electron microscopy (SEM) was used to observe the cross-sectional microstructure of BR and GBR grains at different germination stages. BR and GBR samples were freeze-dried, sectioned perpendicular to the longitudinal axis, mounted on aluminum stubs using conductive carbon tape, and sputter-coated with a thin gold layer. Imaging was performed using a field-emission SEM (Zeiss Gemini SEM 360, Carl Zeiss Co., Ltd., Oberkochen, Germany) at an accelerating voltage of 5 kV. Images were obtained at magnifications of 300× and 1000×, with working distances ranging from 7.1 to 8.7 mm.

### 2.5. Evaluation of Cholesterol-Lowering Effects and Identification of Active Compounds in BR and GBR

#### 2.5.1. Cell Culture and Cytotoxicity Assessment

Human HepG2 cells were obtained from the Shanghai Institute of Cell Biology, Chinese Academy of Sciences (Shanghai, China). Cells were maintained in Dulbecco’s modified Eagle medium (DMEM) supplemented with 10% fetal bovine serum (FBS) and 1% penicillin/streptomycin (100 μg/mL streptomycin and 100 U/mL penicillin) in a humidified incubator at 37 °C with 5% CO_2_.

To determine the non-cytotoxic concentration range for subsequent experiments, the cytotoxic effects of the samples on HepG2 cells were first evaluated using the MTT method described by Li [14]. Briefly, cells were seeded in 96-well plates at a density of 1.5 × 10^4^ cells/well. After 12 h, the cells were treated with various concentrations of the samples for 24 h, followed by incubation with MTT solution (5 mg/mL, 10 μL/well) for 4 h. Subsequently, 150 μL of DMSO was added to each well, and the plate was shaken for 10 min at room temperature. Absorbance was measured at 490 nm using a Bio Tek Epoch 2 microplate reader to determine cell viability.

#### 2.5.2. Hypercholesterolemic Model Establishment and Intervention

Following the cytotoxicity assessment, the cholesterol-lowering effects of the samples were evaluated in a hypercholesterolemic cell model. When HepG2 cells reached 80–90% confluence, the cells were harvested using 0.25% trypsin and seeded in 6-well plates at a density of 5 × 10^5^ cells per well. After cell attachment, the culture medium was replaced and the cells were assigned to the following groups and treated accordingly. (1) Control group: Cells were treated with the medium containing DMSO (0.15%). (2) Model group: Cells were treated with a high-cholesterol induction solution prepared with a final concentration of 10 μg/mL of cholesterol and 1 μg/mL of 25-hydroxycholesterol as previously reported [15]. (3) Treatment groups: Based on Group (2), cells were treated with different concentrations (selected based on the MTT results) of intervention substances (dissolved in DMSO). The culture was harvested after 24 h of incubation.

For lipid content analysis, cellular levels of TC, TG, HDL-C, and LDL-C were measured using commercial assay kits (Nanjing Jiancheng Biotechnology Research Institute Co., Ltd., Nanjing, China) according to the manufacturer’s instructions.

#### 2.5.3. LC-MS Analysis

UPLC-QTOF-MS was used as the analytical platform for untargeted metabolomic profiling of methanolic extracts from BR and GBR. Separation was achieved on a BEH C18 column (100 × 2.1 mm, 1.7 μm) using a gradient of 0.1% formic acid in water (mobile phase A) and 0.1% formic acid in acetonitrile (mobile phase B) at a flow rate of 0.3 mL/min. Each cycle included 3 min of equilibration, with an injection volume of 1 μL. Specific elution gradient of the mobile phase and mass spectrometric conditions are shown in Tables S1 and S2.

#### 2.5.4. Screening of Active Compounds in GBR

Untargeted metabolomic profiling was performed using UPLC-QTOF-MS to compare methanolic extracts of BR and GBR. A total of 626 metabolites were detected. Differential metabolites between the two groups were screened according to the criteria of VIP > 1, *p* < 0.05, and FC > 2 or <0.5. Among these differential metabolites, the top 20 compounds ranked by VIP value were selected for further evaluation.

Spearman correlation analysis was then performed between the abundance of these top 20 metabolites and the reduction rate of intracellular TC in the cholesterol-induced HepG2 cell model, and candidate metabolites were prioritized according to their correlation coefficients with cholesterol-related functional indicators. In addition, based on a modified method described by Wu et al. [16], absorption-related parameters, including oral bioavailability (OB), human intestinal absorption (HIA), and Caco-2 permeability, were evaluated only as supportive information to assist candidate prioritization, rather than as definitive criteria for identifying bioactive compounds in the food matrix. Relevant literature regarding cholesterol-lowering or lipid-regulating activity was also taken into consideration. Candidate compounds prioritized through this workflow were then subjected to subsequent validation experiments.

### 2.6. Quantitative Analysis by HPLC

HPLC was used for targeted quantification of selected phenolic compounds. The analysis was performed on a Shimadzu LC-20 A system equipped with a Waters C18 column (4.6 × 250 mm, 5 μm) and an SPD-20 A photo-diode array detector. The chromatographic conditions, adapted from Zhang et al. [17], utilized a binary solvent system for elution: mobile phase A was aqueous 0.1% acetic acid and mobile phase B was 0.1% acetic acid in methanol. The gradient elution procedure is shown in Table S3. Quantification was performed using the external standard method, with p-coumaric acid as the reference standard. Chromatographic detection was carried out at 320 nm, and quantification was based on peak area. The calibration curve for p-coumaric acid was established as follows: y = 59778x − 42868 (R^2^ = 0.9995).

### 2.7. In Vitro Evaluation of the Cholesterol-Lowering Effects and Underlying Mechanisms of the Active Compound

#### 2.7.1. In Vitro Cell Experiments

The assays for cell viability and cholesterol-related parameters (TC, TG, LDL-C and HDL-C) were performed according to the procedure described in Section 2.5.1 and Section 2.5.2.

#### 2.7.2. Molecular Mechanism Verification

RNA isolation and quantitative real-time PCR (qRT-PCR) analysis were performed according to the method described by Liu et al. [18]. Total RNA was isolated from HepG2 cells using TRIzol reagent (Invitrogen, Carlsbad, CA, USA) following the manufacturer’s protocol. RNA concentration and purity were determined spectrophotometrically using a Nano Drop 2000 system (Thermo Fisher Scientific, Waltham, MA, USA). Complementary DNA (cDNA) was synthesized from 1 μg total RNA using the Prime Script RT reagent kit with gDNA Eraser (Takara Bio, Shiga, Japan). qRT-PCR amplification was performed in triplicate using the Quant Studio 5 Real-Time PCR System (Applied Biosystems, Foster City, CA, USA) with Power Up SYBR Green Master Mix (Applied Biosystems). The thermal cycling conditions consisted of an initial denaturation at 95 °C for 10 min, followed by 40 cycles of 95 °C for 15 s and 60 °C for 1 min. β-actin served as the endogenous control for normalization, and relative gene expression levels were calculated using the comparative 2^−ΔΔCt^ method. All primer sequences used for qRT-PCR are listed in Supplementary Table S4.

### 2.8. Statistical Analysis

All quantitative data are expressed as mean ± standard deviation (SD). At least three independent experiments were conducted for each experimental setup, and representative data are shown. Statistical analyses were performed using GraphPad Prism9.0 and SPSS Statistics26. Normality was assessed using the Shapiro–Wilk test, and homogeneity of variance was evaluated using Levene’s test before parametric analysis. Comparisons among three or more groups were performed using one-way ANOVA followed by Tukey’s multiple comparison test. A *p* value < 0.05 was considered statistically significant, and significance levels were indicated as * for *p* < 0.05, ** for *p* < 0.01, and *** for *p* < 0.001. For multiple-group comparisons, different lowercase letters above the bars indicate significant differences between groups (*p* < 0.05), whereas the same lowercase letter denotes no significant difference.

For metabolomics analysis, five independent biological replicates were used for each group. Mass spectrometry data were processed using Progenesis QI3.0, and subsequent visualization and statistical analyses were performed using the Bio Ladder platform, Metware Cloud, and MetaboAnalyst. Principal component analysis (PCA) was conducted to visualize the overall metabolic variation and sample separation between BR and GBR. Hierarchical cluster analysis (HCA) was used to evaluate similarity patterns among samples and metabolites and to generate the clustering heatmap. In addition, orthogonal partial least squares discriminant analysis (OPLS-DA) was performed to further distinguish the metabolic profiles of BR and GBR. Differential metabolite analysis was then conducted, and volcano plots were used to display the upregulated and downregulated metabolites.

## 3. Results and Discussion

### 3.1. Changes in Phenolic and Flavonoid Contents and Antioxidant Activities During Germination

Based on the rationale described in the Introduction, the four selected BR cultivars were further compared for their germination-related changes in phenolic accumulation, antioxidant activity, and cholesterol-lowering potential.

**Figure 1:**
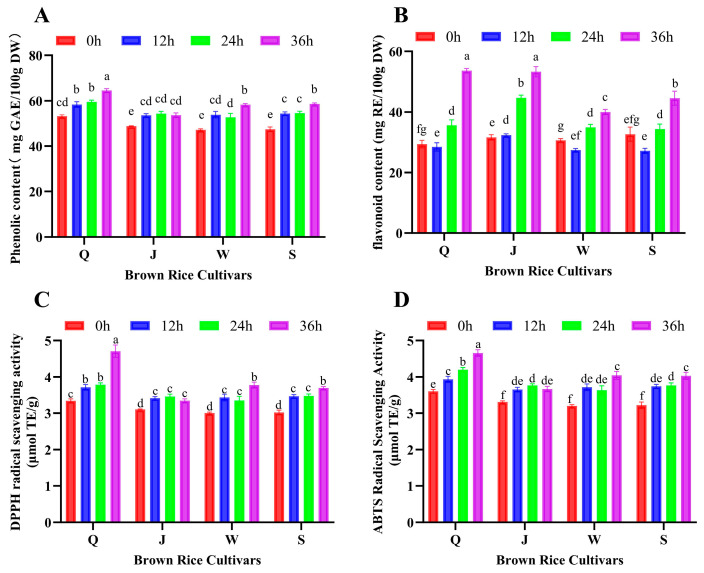
Polyphenol and flavonoid content and antioxidant activity of extracts from various BR cultivars at different germination times. (**A**) Polyphenol content in BR extracts across cultivars and germination times. (**B**) Flavonoid content in BR extracts across cultivars and germination times. (**C**) DPPH scavenging activity of BR extracts from different cultivars and germination times. (**D**) ABTS scavenging activity of BR extracts from different cultivars and germination times. Data are presented as mean ± SD (*n* = 3). Different lowercase letters indicate significant differences among groups (*p* < 0.05).

The germination performance of the four BR cultivars at different germination stages is summarized in Supplementary Table S5. Under the present conditions, the germination rate increased progressively with germination time, with the highest germination level being observed at 36 h. Consistent with this trend, germination significantly affected the TPC of the four BR cultivars (*p* < 0.05) (Figure 1A). For all cultivars, TPC increased after germination compared with the ungerminated grains (0 h), and the most pronounced increases were generally observed at 36 h. Cultivar Q exhibited the largest increase in TPC during germination, whereas cultivar J showed a more moderate response, with similar values from 12 to 36 h. Overall, these results indicate that short-term germination can effectively enhance the accumulation or release of phenolic compounds in BR, although the magnitude of this response was clearly cultivar dependent. A similar pattern was observed for TFC (Figure 1B). Germination for 24–36 h significantly increased TFC in all four cultivars (*p* < 0.05), with cultivar Q showing the highest level at 36 h. The stronger response of cultivar Q may be associated with genotype-dependent differences in the initial distribution of phenolic compounds and in the metabolic activation induced by germination. Since the functional quality of germinated brown rice is influenced by both genotype and germination conditions, and rice polyphenols exhibit substantial genetic diversity among cultivars, cultivar Q may possess a greater capacity to accumulate or release phenolic and flavonoid compounds during germination [19,20].

The changes in antioxidant capacity, evaluated by DPPH and ABTS radical scavenging assays, followed the same general pattern as phenolics and flavonoids (Figure 1C,D). For each cultivar, DPPH and ABTS scavenging activities increased significantly with germination time (*p* < 0.05), and the highest activities were usually recorded at 36 h. Cultivar Q again showed the greatest improvement, while J, W and S displayed smaller but still significant enhancements relative to 0 h (*p* < 0.05). To further examine the relationship between phenolic accumulation and antioxidant capacity, correlation analysis was performed between TPC and DPPH/ABTS radical scavenging activities. As shown in Supplementary Figure S2, TPC was positively correlated with DPPH and ABTS radical scavenging activities, suggesting that the accumulation of phenolic compounds may contribute, at least in part, to the enhanced antioxidant capacity of GBR during germination.

Taken together, these findings align with previous reports establishing strong correlations between phenolic content and antioxidant activities [21], suggesting that phenolic compounds may be important contributors to the antioxidant effects observed during germination.

### 3.2. SEM Analysis

**Figure 2:**
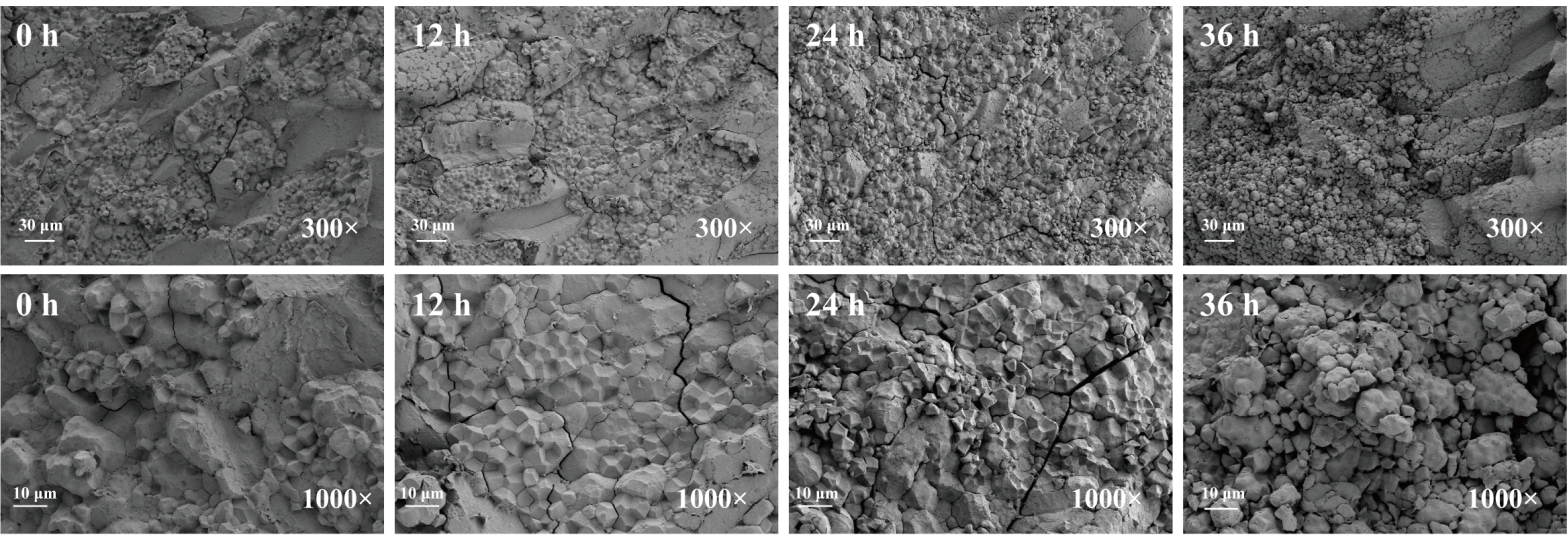
SEM images of cross-sections of BR grains with different germination times.

The microstructural changes in BR during germination can be directly observed from the SEM cross-sectional images. As shown in Figure 2, the outer surface of BR gradually became looser and its structure became increasingly disrupted during the germination process. Compared to ungerminated brown rice, more pronounced structural breakdown and porosity were observed after 36 h of germination. This may be due to the partial degradation of protein bodies following germination, causing the endosperm to develop a porous, honeycomb-like structure [22].

Although SEM alone cannot directly demonstrate changes in the composition or bioactivity of phenolic compounds, these structural changes may facilitate the release of bound phenolic compounds during the germination process [17].

### 3.3. Effects of BR and Germinated BR on Cell Viability and Lipid Metabolism in High-Cholesterol HepG2 Cells

**Figure 3:**
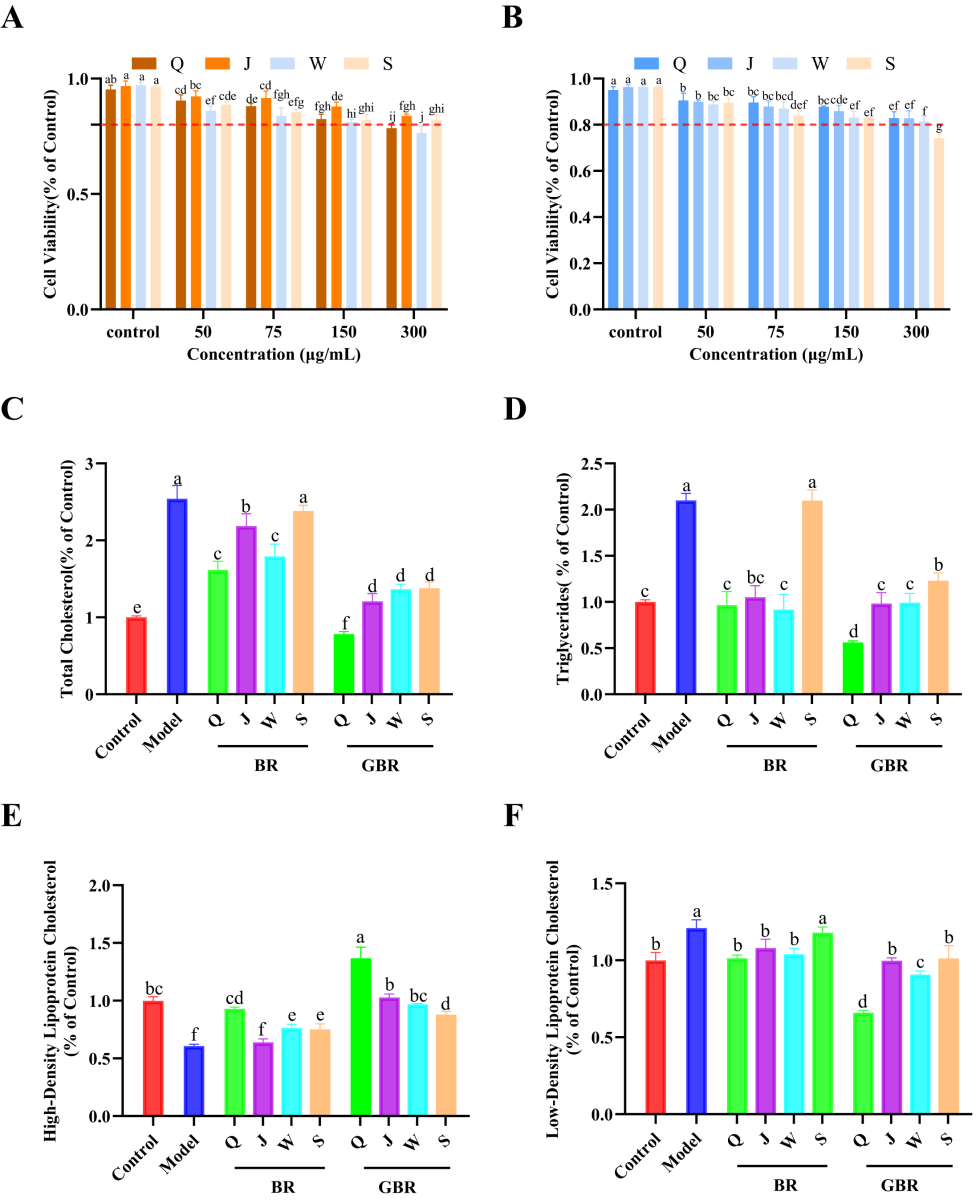
Effects of different concentrations of BR and GBR on the viability of HepG2 cells and its regulatory role in the HepG2 hypercholesterolemia cell model. (**A**) Effect of BR on cell viability. (**B**) Effect of GBR on cell viability. (**C**) Intracellular TC content. (**D**) intracellular TG content. (**E**) intracellular LDL-C content. (**F**) intracellular HDL-C content. Data are presented as mean ± SD (*n* = 3). Different lowercase letters indicate significant differences among groups (*p* < 0.05).

Based on the previous experiments, all four BR cultivars exhibited the highest contents of total phenolics and flavonoids, as well as the strongest antioxidant activities, after 36 h of germination. Therefore, a germination time of 36 h was selected for subsequent experiments. As shown in Figure 3A,B, extracts from both BR and GBR for 36 h exhibited low cytotoxicity toward HepG2 cells. In the concentration range of 50–150 μg/mL, cell viability remained above 80% of the untreated control for all four cultivars (Q, J, W and S). Based on the MTT assay, 150 μg/mL was selected as the working concentration for subsequent functional evaluation because it showed no obvious cytotoxicity and produced measurable effects in the in vitro screening system.

High-cholesterol stimulation markedly disturbed lipid homeostasis in HepG2 cells (Figure 3C–F). Compared with the control group, the model group showed significant increases in intracellular TC, TG and LDL-C, while HDL-C was significantly reduced (all *p* < 0.05), confirming the successful establishment of a cholesterol-induced HepG2 cell model.

Treatment with BR extracts from the four cultivars partially reversed these lipid abnormalities. All BR groups significantly lowered TC and TG levels relative to the model group (*p* < 0.05), although the values remained higher than those of the control. Similar tendencies were observed for LDL-C, which was modestly reduced by BR treatment, whereas HDL-C was restored to or slightly above the control level in some cultivars (Figure 3C–F). These data suggest that BR possesses a basal hypolipidemic effect in hepatocytes, but the magnitude of improvement is cultivar-dependent.

In contrast, GBR extracts exerted a more pronounced lipid-regulating effect. For all four cultivars, germination for 36 h led to significantly lower TC, TG and LDL-C levels (*p* < 0.05) compared with their corresponding BR groups, accompanied by a further increase in HDL-C. Notably, GBR from cultivar Q produced TC and TG levels close to those of the control and markedly elevated HDL-C above the control value, indicating the strongest protective effect among the tested samples. Overall, these results demonstrate that germination substantially enhances the ability of BR to ameliorate cholesterol-induced lipid accumulation in HepG2 cells, which is consistent with the increased bioactive compound and antioxidant levels observed in GBR grains [21].

### 3.4. Component Identification of BR and GBR

Based on the previous experimental results, cultivar Q exhibited the most pronounced cholesterol-modulating effect among the tested cultivars. Therefore, Q samples before and after germination were selected for subsequent metabolomic analysis. There were clear differences in the cholesterol-lowering effects of BR extract before and after germination, reflecting the dynamic changes in active components during germination [23].

**Figure 4:**
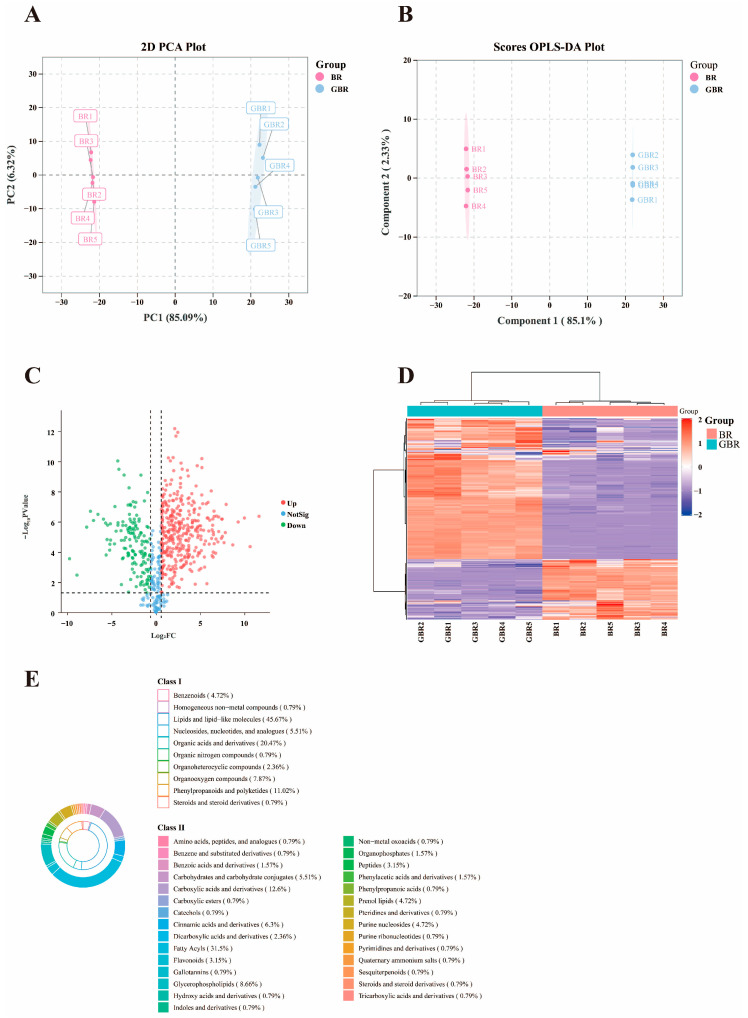
Untargeted metabolite profiling of cultivar Q before and after germination. (**A**) Score plots of PCA among the two groups. (**B**) Score plots of OPLS-DA among the two groups. (**C**) Volcano map of differential substances. (**D**) Heat map of differential substances. (**E**) Classification of differential substances.

Figure 4 summarizes the untargeted metabolomic differences between BR and GBR. A total of 626 metabolites were detected in BR and GBR samples by untargeted metabolomic profiling. In the unsupervised PCA score plot (Figure 4A), the two groups were clearly separated along PC1. PC1 and PC2 explained 85.09% and 6.32% of the total variance, respectively, and together accounted for 91.41% of the overall variance, indicating that the first two principal components effectively captured the major metabolic differences between the two groups. Biological replicates within each group clustered tightly. This indicates that germination induced a systematic shift in the overall metabolite profile. Consistently, the supervised OPLS-DA model (Figure 4B) showed a distinct discrimination between BR and GBR on the predictive component, further confirming that germination was the main source of variation in the dataset. Figure S1 showed that the model parameters R2Y = 1 and Q2 = 0.999, and the permutation test *p* value was <0.001, confirming that the model had good predictive ability [24].

The volcano plot (Figure 4C) revealed a large number of differential metabolites between BR and GBR. Both up-regulated and down-regulated metabolites were observed, indicating that germination activates some metabolic pathways while suppressing others. Hierarchical clustering of the differential metabolites (Figure 4D) produced two major clusters corresponding to BR and GBR, again demonstrating a consistent and group-specific metabolic pattern across samples.

**Figure 5:**
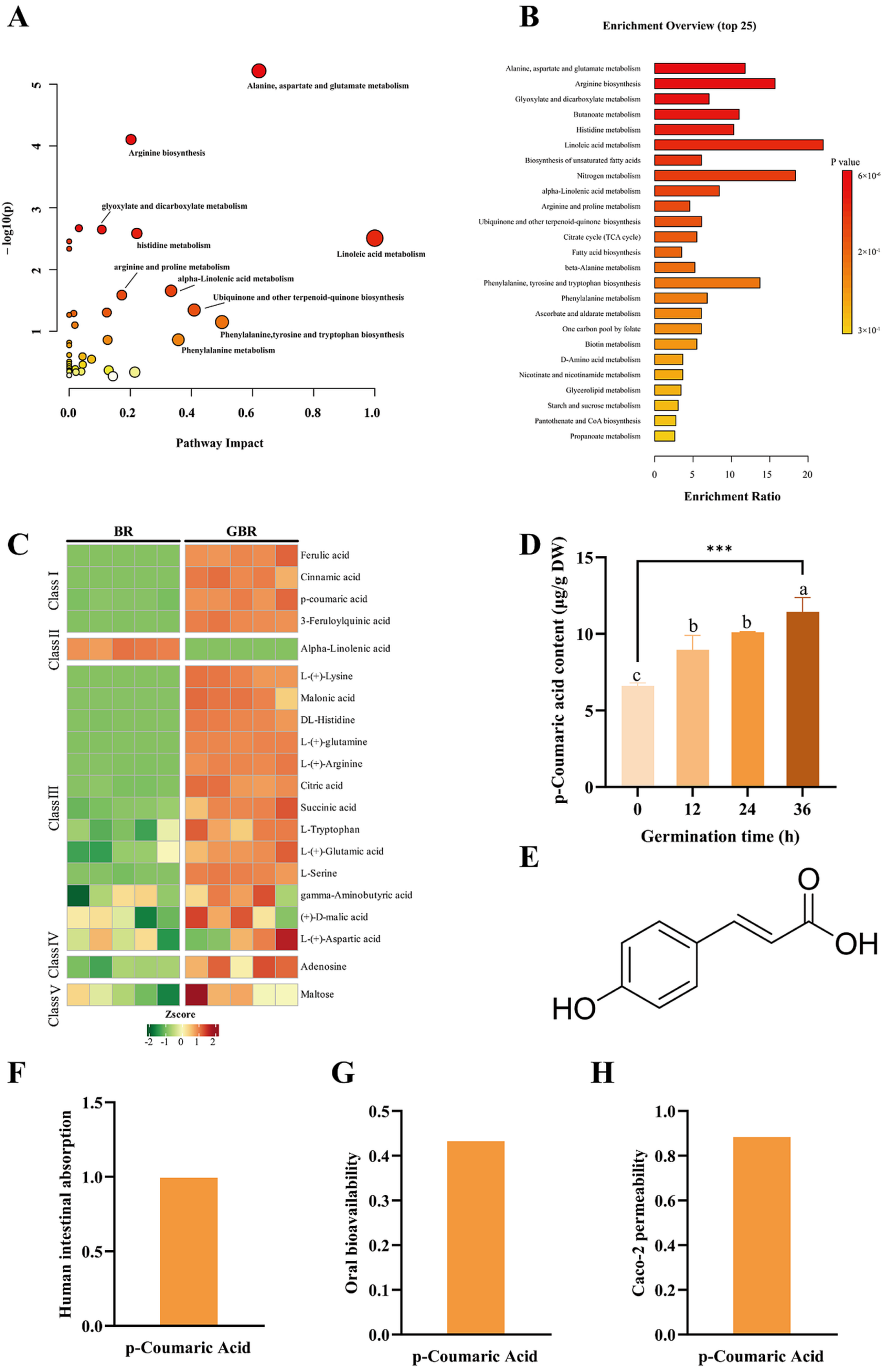
Untargeted metabolite profiling of metabolic changes in cultivar Q before and after germination and screening of candidate active ingredients in Q GBR. (**A**) Pathway analysis of differential metabolites between BR and GBR. (**B**) Differential metabolite enrichment analysis between BR and GBR. (**C**) The top 20 substances in BR and GBR with VIP > 1. (**D**) The content of p-coumaric acid in brown rice at different germination times. (**E**) Chemical structures of p-coumaric acid. (**F**) Human intestinal absorption, (**G**) oral bioavailability and (**H**) Caco-2 permeability of p-coumaric acid. Data are presented as mean ± SD (*n* = 3). Different lowercase letters indicate significant differences among groups (*p* < 0.05). *** indicates a highly significant difference between the indicated groups (*p* < 0.001).

Chemical classification of the discriminant metabolites (Figure 4E) showed that they were mainly distributed in several major classes, including phenolic acids and other phenylpropanoids, flavonoids, amino acid derivatives, organic acids and lipids. The enrichment of phenolic- and flavonoid-type metabolites in GBR is in line with the increased phenolic content and antioxidant activity observed at the macroscopic level, suggesting that germination promotes the biosynthesis or release of these bioactive compounds [25]. Together, these results indicate that germination substantially remodels the metabolome of BR, especially in pathways related to phenolic metabolism and energy utilization, which may underlie the improved nutritional and functional properties of GBR. As shown in Figure 5A,B, the differential metabolites between BR and GBR are predominantly enriched in lipid metabolism–related pathways, as well as in energy, carbohydrate and amino acid metabolism. This pattern suggests a close association between these differential metabolites and lipid metabolic processes [16].

### 3.5. Screening of Cholesterol-Lowering Components in GBR

VIP scores were used as a preliminary screening tool to assess the contribution of individual metabolites to the discrimination between BR and GBR samples. In the present study, differential metabolites were first screened according to the criteria of VIP > 1, *p* < 0.05, and FC > 2 or <0.5. Among these, the top 20 metabolites ranked by VIP value were retained for further evaluation (Figure 5C and Table S6). These metabolites mainly included phenolic acids, amino acid derivatives, organic acids, sugars, and fatty acid-related compounds, indicating that germination induced broad metabolic remodeling in brown rice.

To further identify metabolites potentially associated with cholesterol-lowering activity, Spearman correlation analysis was performed between the abundance of the top 20 differential metabolites and the reduction rate of intracellular TC in the cholesterol-induced HepG2 cell model. Several metabolites showed positive correlations with the TC reduction rate (Table S6), suggesting a possible association with the cholesterol-lowering activity of GBR. Among these candidates, phenolic acids were particularly notable because they were not only significantly enriched after germination but were also closely related to the phenylpropanoid biosynthesis and phenylalanine metabolism pathways (Figure 5A,B), which are known to contribute to the formation of bioactive phenolic compounds.

Among the candidate metabolites, p-coumaric acid showed a pronounced increase during germination and reached its highest level at 36 h (Figure 5D, *p* < 0.05). This change was consistent with the overall increases in total phenolic content, total flavonoid content, and antioxidant activity observed in GBR. In addition, p-coumaric acid exhibited a favorable combination of high VIP ranking, and a relatively strong positive correlation with the intracellular TC reduction rate compared with many other candidate metabolites. Previous studies have also reported lipid-regulating or metabolic protective effects of p-coumaric acid, further supporting its biological relevance as a candidate compound [26].

To provide additional supportive information for candidate prioritization, absorption-related parameters, including oral bioavailability (OB), human intestinal absorption (HIA), and Caco-2 permeability, were also evaluated (Figure 5E–H). These parameters suggested that p-coumaric acid possesses acceptable absorption-related properties. Taken together, the metabolomics screening, correlation analysis, literature support, and auxiliary absorption-related evaluation identified p-coumaric acid as a candidate bioactive compound in GBR and a reasonable target for subsequent validation experiments [16].

### 3.6. Verification Experiment on p-Coumaric Acid

**Figure 6:**
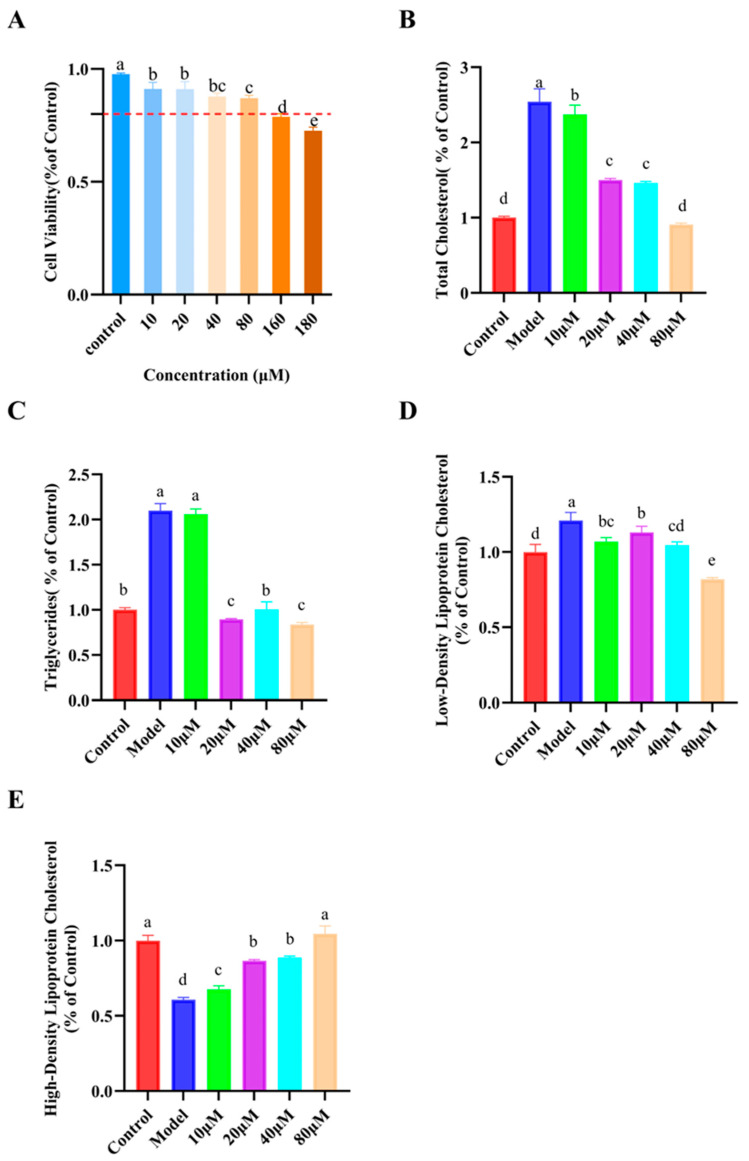
Effects of p-coumaric acid on intracellular lipid levels in the cholesterol-induced HepG2 cell model. (**A**) Effect of p-coumaric acid on cell viability. (**B**) Intracellular TC content. (**C**) intracellular TG content. (**D**) intracellular LDL-C content. (**E**) intracellular HDL-C content. Data are presented as mean ± SD (*n* = 3). Different lowercase letters indicate significant differences among groups (*p* < 0.05).

To evaluate the safety of p-coumaric acid, HepG2 cells were co-incubated with 0 to 180 μM of p-coumaric acid for 24 h. The concentration range of p-coumaric acid was selected based on preliminary range-finding experiments to cover non-cytotoxic but biologically responsive concentrations in the in vitro model. Figure 6 shows the effects of p-coumaric acid on cell viability and cholesterol-related parameters in HepG2 cells. As shown in Figure 6A, treatment with 10–80 μM p-coumaric acid caused only a slight, though statistically detectable, reduction in cell viability compared with the control, whereas 80–180 μM led to a more pronounced decrease. However, cell viability remained above 80% at all tested concentrations, indicating that p-coumaric acid exhibited no obvious cytotoxicity within this range and was suitable for subsequent functional assays.

In the cholesterol-overloaded HepG2 model, intracellular total TC and TG were markedly elevated relative to the control group, while LDL-C increased and HDL-C decreased, confirming successful establishment of the hypercholesterolemic state (Figure 6B–E). p-Coumaric acid treatment significantly improved these lipid abnormalities (*p* < 0.05) in a concentration-dependent manner. Compared with the model group, 10–80 μM p-coumaric acid progressively reduced TC and TG levels, with the greatest decreases observed at 80 μM (Figure 6B,C). A similar trend was found for LDL-C (Figure 6D), whereas HDL-C was partially restored at 10–40 μM and almost returned to control levels at 80 μM (Figure 6E).

While these results support the biological relevance of p-coumaric acid in the in vitro model, its contribution to the activity of the crude GBR extract should still be interpreted with caution. Based on its quantified content in the 36 h GBR sample and the working concentration of GBR extract used in the cell assay, the estimated concentration of p-coumaric acid in the culture system was substantially lower than the concentration range at which p-coumaric acid showed biological activity in the validation experiments. This suggests that p-coumaric acid alone may not be sufficient to fully account for the observed bioactivity of the crude extract. Rather, it is more likely that p-coumaric acid represents one of the contributing components, while other bioactive constituents in GBR, as well as possible synergistic interactions among them, may also participate in the cholesterol-lowering effect [27].

### 3.7. Effects of p-Coumaric Acid on the Expression of Hepatic Lipid-Metabolism Genes in Hypercholesterolemic HepG2 Cells

**Figure 7:**
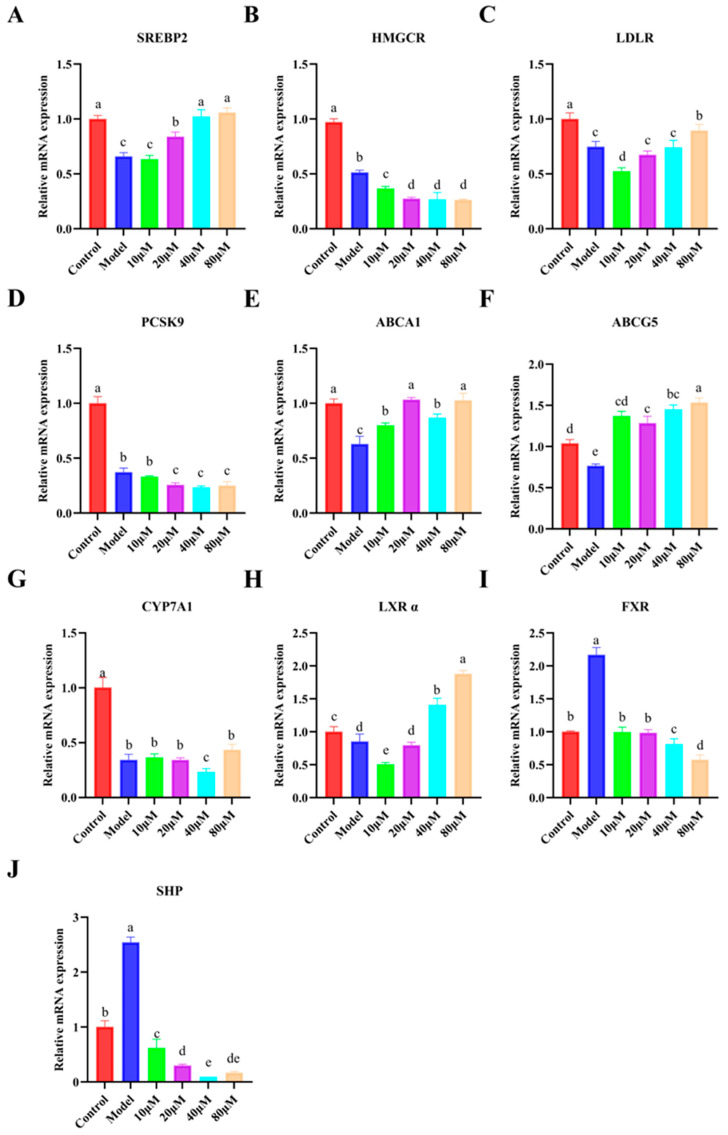
Effects of p-coumaric acid on the mRNA expression of cholesterol metabolism-related genes in the cholesterol-induced HepG2 cell model. (**A**) SREBP2; (**B**) HMGCR; (**C**) LDLR; (**D**) PCSK9; (**E**) ABCA1; (**F**) ABCG5; (**G**) CYP7A1; (**H**) LXR α; (**I**) FXR; (**J**) SHP. Data are presented as mean ± SD (*n* = 3). Different lowercase letters indicate significant differences among groups (*p* < 0.05).

To further explore the possible transcriptional basis underlying the cholesterol-lowering effect of p-coumaric acid, the mRNA expression levels of genes related to cholesterol synthesis, uptake, efflux, and bile acid synthesis were analyzed in the cholesterol-induced HepG2 cell model (Figure 7). Compared with the control group, cholesterol induction markedly altered the expression profile of multiple lipid-related genes, indicating that cholesterol homeostasis was substantially disturbed in the model cells. Specifically, the model group showed decreased SREBP2, HMGCR, PCSK9, ABCA1, ABCG5, CYP7A1, and LXR α expression, whereas LDLR, FXR, and SHP were upregulated (*p* < 0.05). Following p-coumaric acid treatment, several of these transcriptional changes were partially or progressively reversed, although the degree of recovery varied among genes.

With respect to cholesterol synthesis- and uptake-related genes, p-coumaric acid treatment significantly modulated the mRNA expression of SREBP2, HMGCR, LDLR, and PCSK9. As shown in Figure 7A–D, HMGCR and PCSK9 expression decreased in a dose-dependent manner after treatment with 10–80 μM p-coumaric acid, while SREBP2 and LDLR tended to return toward the control level, particularly at higher concentrations. Since SREBP2 is a central transcriptional regulator of cholesterol homeostasis and PCSK9 is closely involved in LDLR regulation [28,29,30], these changes suggest that p-coumaric acid may be associated with the modulation of cholesterol synthesis and LDL handling at the transcriptional level in the present in vitro system. At the same time, the reduction in HMGCR expression is consistent with a potential suppression of cholesterol biosynthesis-related signaling.

In addition, p-coumaric acid treatment influenced the expression of LXRα, ABCA1, and ABCG5, which are closely related to cholesterol transport and efflux (Figure 7E–H). Compared with the model group, LXR α and ABCG5 were progressively upregulated by p-coumaric acid, and ABCA1 also showed a recovery trend, especially at 40 and 80 μM. Because ABCA1 is a key transporter involved in reverse cholesterol transport and LXRα is an important upstream regulator of efflux-related genes [31,32], these results suggest that p-coumaric acid may contribute to a cellular state more favorable for cholesterol efflux.

The expression patterns of CYP7A1, FXR, and SHP further suggest that bile acid synthesis-related signaling may also be involved in the response to p-coumaric acid (Figure 7G–J). In the model group, CYP7A1 and LXRα were suppressed, whereas FXR and SHP were elevated, indicating an altered transcriptional state associated with bile acid metabolism. P-coumaric acid treatment partially restored CYP7A1 expression while reducing FXR and SHP mRNA levels in a concentration-dependent manner. Since CYP7A1 is the rate-limiting enzyme in bile acid synthesis, whereas FXR and SHP are important regulators of this pathway [33,34], these results imply that p-coumaric acid may be associated with the transcriptional regulation of cholesterol conversion to bile acids in cholesterol-induced HepG2 cells. Nevertheless, because neither bile acid production nor protein activity were measured, this interpretation should still be considered preliminary.

Taken together, the results shown in Figure 7 indicate that p-coumaric acid treatment was accompanied by coordinated changes in the mRNA expression of multiple cholesterol metabolism-related genes in the cholesterol-induced HepG2 model. These changes involved genes associated with cholesterol synthesis (SREBP2, HMGCR), uptake and LDL handling (LDLR, PCSK9), cholesterol efflux (LXRα, ABCA1, ABCG5), and bile acid synthesis-related signaling (CYP7A1, FXR, SHP). Therefore, the cholesterol-lowering effect of p-coumaric acid observed in the present study may be associated with broad transcriptional modulation of cholesterol homeostasis.

## 4. Conclusions

This study demonstrated that after 36 h of germination treatment on four types of BR, the content of polyphenols, flavonoids, and antioxidant activity in their extracts all increased, although the degree of response varied among different cultivars. The GBR extracts also improved the lipid-related indicators in a cholesterol-induced HepG2 cell model, showing a decrease in TC, TG and LDL-C, and an increase in HDL-C. Based on untargeted metabolomics analysis and subsequent verification, p-coumaric acid was identified as a candidate bioactive compound that improves cholesterol metabolism. Further analysis indicated that this effect may be related to the regulation of mRNA expression of genes involved in cholesterol metabolism, including HMGCR, LDLR, and CYP7A1. Overall, these findings suggest that germination enhances the biological activity of BR in a cultivar-dependent manner, and provide supporting evidence for the potential application of GBR as a functional food component for lipid management.

Several limitations of the present study should be noted. The mechanistic findings were obtained in an in vitro model using cholesterol-induced HepG2 cells, which cannot fully replicate the complexity of cholesterol metabolism in vivo. In addition, although this study assessed changes in mRNA expression, it did not measure the corresponding protein levels or enzyme activities of the targets under investigation. While p-coumaric acid was identified as a candidate bioactive compound, the potential contribution of other active components in GBR cannot be ruled out. Anti-nutritional factors were also not evaluated in the present study, and future work should include their assessment. In addition, although the extraction conditions were selected based on the experimental protocol used in this study, the extraction yield and recovery efficiency of phenolic compounds were not systematically evaluated. Moreover, the physiological relevance of the selected in vitro extract concentration remains to be further evaluated in vivo.

## Data Availability

The original contributions presented in this study are included in the article/Supplementary Materials. Further inquiries can be directed to the corresponding authors.

## Supplementary Materials

The following supporting information can be downloaded at: https://www.mdpi.com/article/10.3390/foods15081344/s1, Figure S1: OPLS-DA score plot and permutation test results for the BR and GBR groups; Figure S2: correlations between total phenolic content and DPPH/ABTS radical scavenging activities in the four BR cultivars; Figure S3: morphological observations of cholesterol-induced HepG2 cells after 24 h treatment with different concentrations of p-coumaric acid; Table S1: UPLC gradient elution program; Table S2: mass spectrometry parameters; Table S3: HPLC gradient elution program; Table S4: primer sequences for qRT-PCR; Table S5: germination rates of four rice cultivars at different germination times; Table S6: top 20 metabolites ranked by VIP values.

## References

[1] Centonze G., Natalini D., Piccolantonio A., Salemme V., Morellato A., Arina P., Riganti C., Defilippi P. (2022). Cholesterol and its derivatives: Multifaceted players in breast cancer progression. Front. Oncol..

[2] Borén J., Chapman M.J., Krauss R.M., Packard C.J., Bentzon J.F., Binder C.J., Daemen M.J., Demer L.L., Hegele R.A., Nicholls S.J. (2020). Low-Density Lipoproteins Cause Atherosclerotic Cardiovascular Disease: Pathophysiological, Genetic, and Therapeutic Insights: A Consensus Statement from the European Atherosclerosis Society Consensus Panel. Eur. Heart J..

[3] Wu Y.-R., Li L., Sun X.-C., Wang J., Ma C.-Y., Zhang Y., Qu H.-L., Xu R.-X., Li J.-J. (2021). Diallyl Disulfide Improves Lipid Metabolism by Inhibiting PCSK9 Expression and Increasing LDL Uptake via PI3K/Akt-SREBP2 Pathway in HepG2 Cells. Nutr. Metab. Cardiovasc. Dis..

[4] Tammi R., Männistö S., Maukonen M., Kaartinen N.E. (2023). Whole Grain Intake, Diet Quality and Risk Factors of Chronic Diseases: Results from a Population-Based Study in Finnish Adults. Eur. J. Nutr..

[5] Cho D.-H., Lim S.-T. (2016). Germinated Brown Rice and Its Bio-Functional Compounds. Food Chem..

[6] Luo X., Tao Y., Han Y., Wang P., Li D. (2023). Effect of Static Magnetic Field Treatment on γ-Aminobutyric Acid Content and Sensory Characteristics of Germinated Brown Rice Cake. Food Chem..

[7] Fukushima A., Uchino G., Akabane T., Aiseki A., Perera I., Hirotsu N. (2020). Phytic Acid in Brown Rice Can Be Reduced by Increasing Soaking Temperature. Foods.

[8] Hao C.-L., Lin H.-L., Ke L.-Y., Yen H.-W., Shen K.-P. (2019). Pre-Germinated Brown Rice Extract Ameliorates High-Fat Diet-Induced Metabolic Syndrome. J. Food Biochem..

[9] Zhou C., Zhou Y., Hu Y., Li B., Zhang R., Zheng K., Liu J., Wang J., Zuo M., Liu S. (2021). Integrated Analysis of Metabolome and Volatile Profiles of Germinated Brown Rice from the Japonica and Indica Subspecies. Foods.

[10] Dai W., Xie D., Lu M., Li P., Lv H., Yang C., Peng Q., Zhu Y., Guo L., Zhang Y. (2017). Characterization of White Tea Metabolome: Comparison against Green and Black Tea by a Nontargeted Metabolomics Approach. Food Res. Int..

[11] Wu N.-N., Li R., Li Z.-J., Tan B. (2022). Effect of Germination in the Form of Paddy Rice and Brown Rice on Their Phytic Acid, GABA, γ-Oryzanol, Phenolics, Flavonoids and Antioxidant Capacity. Food Res. Int..

[12] Chang X., Ye Y., Pan J., Lin Z., Qiu J., Guo X., Lu Y. (2018). Comparative Assessment of Phytochemical Profiles and Antioxidant Activities in Selected Five Varieties of Wampee (*Clausena lansium*) Fruits. Int. J. Food Sci. Technol..

[13] Wang B., Nie C., Li T., Zhao J., Fan M., Li Y., Qian H., Wang L. (2022). Effect of Boiling and Roasting on Phenolic Components and Their Bioaccessibilities of Highland Barley. Food Res. Int..

[14] Li X., Guo J., Liang N., Jiang X., Song Y., Ou S., Hu Y., Jiao R., Bai W. (2018). 6-Gingerol Regulates Hepatic Cholesterol Metabolism by Up-Regulation of LDLR and Cholesterol Efflux-Related Genes in HepG2 Cells. Front. Pharmacol..

[15] Meng C., Zhou L., Huang L., Gu Q., Du X., Wang C., Liu F., Xia C. (2024). Chlorogenic Acid Regulates the Expression of NPC1L1 and HMGCR through PXR and SREBP2 Signaling Pathways and Their Interactions with HSP90 to Maintain Cholesterol Homeostasis. Phytomedicine.

[16] Wu X., Li B., Lu H., Ling X., Hu Z., Luo Y., Qin D., Yang F., Tang Y., Xie T. (2023). Parboiled Rice Extracts Ameliorate Oleic Acid-Induced Steatosis of HepG2 Cell and Its Molecular Mechanism. J. Funct. Foods.

[17] Zhang H., Fan M., Qian H., Wang L., Li Y. (2025). Boiling Outperforms Other Thermal Treatments in Enhancing Bioaccessibility and Absorption of Ferulic Acid in Triticale Bran for Aging Populations. Food Biosci..

[18] Liu J., Li Y., Sun C., Liu S., Yan Y., Pan H., Fan M., Xue L., Nie C., Zhang H. (2020). Geniposide Reduces Cholesterol Accumulation and Increases Its Excretion by Regulating the FXR-Mediated Liver-Gut Crosstalk of Bile Acids. Pharmacol. Res..

[19] Shao Y., Bao J. (2015). Polyphenols in Whole Rice Grain: Genetic Diversity and Health Benefits. Food Chem..

[20] Cáceres P.J., Martínez-Villaluenga C., Amigo L., Frias J. (2014). Maximising the Phytochemical Content and Antioxidant Activity of Ecuadorian Brown Rice Sprouts through Optimal Germination Conditions. Food Chem..

[21] Sumczynski D., Kotásková E., Orsavová J., Valášek P. (2017). Contribution of Individual Phenolics to Antioxidant Activity and In Vitro Digestibility of Wild Rices (*Zizania aquatica* L.). Food Chem..

[22] Yan J., Zhu Y., Hou Y., Luo Z., Li J., Luo W., Zhang Y., Guan X. (2025). A Combination of Germination and Moderate Milling Improves the Textural Quality and Alters Starch Digestion Characteristics of Brown Rice. Int. J. Biol. Macromol..

[23] Ren C., Lu S., Guan L., Hong B., Zhang Y., Huang W., Li B., Liu W., Lu W. (2022). The Metabolomics Variations among Rice, Brown Rice, Wet Germinated Brown Rice, and Processed Wet Germinated Brown Rice. J. Integr. Agric..

[24] Lee S., Lee D.E., Singh D., Lee C.H. (2018). Metabolomics Reveal Optimal Grain Preprocessing (Milling) toward Rice Koji Fermentation. J. Agric. Food Chem..

[25] Barathikannan K., Chelliah R., Vinothkanna A., Prathiviraj R., Tyagi A., Vijayalakshmi S., Lim M.-J., Jia A.-Q., Oh D.-H. (2024). Untargeted Metabolomics-Based Network Pharmacology Reveals Fermented Brown Rice towards Anti-Obesity Efficacy. npj Sci. Food.

[26] Lv R., Liu J., Li S., Gong D., Wang L., Yuan X., Chen X., Li Y. (2025). Discovery of Vitexin, Isovitexin and Catechin as Hypoglycemic Factors in Mung Bean via Metabolomics Combined with In Vitro Experiments. Food Prod. Process. Nutr..

[27] Yoon D.S., Cho S.Y., Yoon H.J., Kim S.R., Jung U.J. (2021). Protective Effects of P-Coumaric Acid against High-Fat Diet-Induced Metabolic Dysregulation in Mice. Biomed. Pharmacother..

[28] Zhu T., Corraze G., Plagnes-Juan E., Skiba-Cassy S. (2020). Cholesterol Metabolism Regulation Mediated by SREBP-2, LXRα and miR-33a in Rainbow Trout (*Oncorhynchus mykiss*) Both In Vivo and In Vitro. PLoS ONE.

[29] Goldstein J.L., DeBose-Boyd R.A., Brown M.S. (2006). Protein Sensors for Membrane Sterols. Cell.

[30] Dubuc G., Chamberland A., Wassef H., Davignon J., Seidah N.G., Bernier L., Prat A. (2004). Statins Upregulate *PCSK9*, the Gene Encoding the Proprotein Convertase Neural Apoptosis-Regulated Convertase-1 Implicated in Familial Hypercholesterolemia. Arterioscler. Thromb. Vasc. Biol..

[31] Guo J., Li Y., Yuan Y., Li X., Li X., Jiang X., Bai W., Jiao R. (2022). Cholesterol-Lowering Activity of 10-Gingerol in HepG2 Cells Is Associated with Enhancing LDL Cholesterol Uptake, Cholesterol Efflux and Bile Acid Excretion. J. Funct. Foods.

[32] Matsuo M. (2022). ABCA1 and ABCG1 as Potential Therapeutic Targets for the Prevention of Atherosclerosis. J. Pharmacol. Sci..

[33] Goodwin B., Jones S.A., Price R.R., Watson M.A., McKee D.D., Moore L.B., Galardi C., Wilson J.G., Lewis M.C., Roth M.E. (2000). A Regulatory Cascade of the Nuclear Receptors FXR, SHP-1, and LRH-1 Represses Bile Acid Biosynthesis. Mol. Cell.

[34] Chambers K.F., Day P.E., Aboufarrag H.T., Kroon P.A. (2019). Polyphenol Effects on Cholesterol Metabolism via Bile Acid Biosynthesis, CYP7A1: A Review. Nutrients.

