# Supplementary material for: Discovery of p-Coumaric Acid as a Candidate Cholesterol-Lowering Factor in Germinated Brown Rice via Untargeted Metabolomics Combined with a Cholesterol-Induced HepG2 Cell Model
Source: Foods. 2026 Apr 13;15(8):1344. doi: 10.3390/foods15081344 (PMC13114526; doi:10.3390/foods15081344)
Supplement: Supplementary file 1 [file foods-15-01344-s001.zip › foods-4193459-supplementary.pdf]

Supplementary material

Table S1 UPLC gradient elution program

Time （min）	Flow rate （mL/min）	A （%）	B （%）
0	0.3	0	100
2	0.3	0	100
6	0.3	100	0
16	0.3	40	60
17	0.3	0	100

Table S2 Mass spectrum parameters

Description	Parameter
cone voltage (V)	35
desolvation gas flow rate (L/h)	600
source temperature (°C)	100
desolvation temperature (°C)	40
collision gas	argon
Scan type (m/z)	50 - 1000

Table S3 HPLC gradient elution program

**Time （min）**	**Flow rate （mL/min）**	**A （%）**	**B （%）**
0	1.0	91	9
11	1.0	86	14
14	1.0	85	15
17	1.0	85	15
24	1.0	83.5	16.5
28	1.0	81	19
30	1.0	75	25
36	1.0	74	26
38	1.0	72	28
41	1.0	65	35
46	1.0	60	40
48	1.0	52	48
53	1.0	47	53
70	1.0	30	70
80	1.0	91	9

Table S4 Primer sequences for qRT-PCR

Gene	Sense Sequence (5’ → 3’)	Antisense Sequence (5’ → 3’)
SREBP2	CTACGGTGCAGACAGTTGCT	CCCACCAGGGTTGGTACTTG
HMGCR	GTCTTGTGGCCAGCACCAAT	TGTTTCGAGCCAGGCTTTCA
LDLR	CAAAGTCTGCAACATGGCTAGA	GTTGTCCAAGCATTCGTTGGTC
ABCA1	ACCCACCCTATGAACAACATGA	GAGTCGGGTAACGGAAACAGG
ABCG5	TGGACCAGGCAGATCCTCAAA	CCGTTCACATACACCTCCCC
CYP7A1	TTACAAGGCGGGACACACAG	CCTCAAGCTCTCTGCCAGTT
PCSK9	ATCCACGCTTCCTGCTGC	CACGGTCACCTGCTCCT
LXRα	GGAGGTACAACCCTGGGAGT	AGCAATGAGCAAGGCAAACT
FXR	TGCAGATCAGACCGTGAATGA	TTGGTTGCCATTTCCGTCAAA
SHP	ACAGCCTGGGACTACACCAC	TGCAAAAACGTGGCATTCTC
β-actin	TGGCACCCAGCACAATGAA	CTAAGTCATAGTCCGCCTAGAA
GAPDH	ATGGGGAAGGTGAAGGTCG	GGGGTCATTGATGGCAACAATA

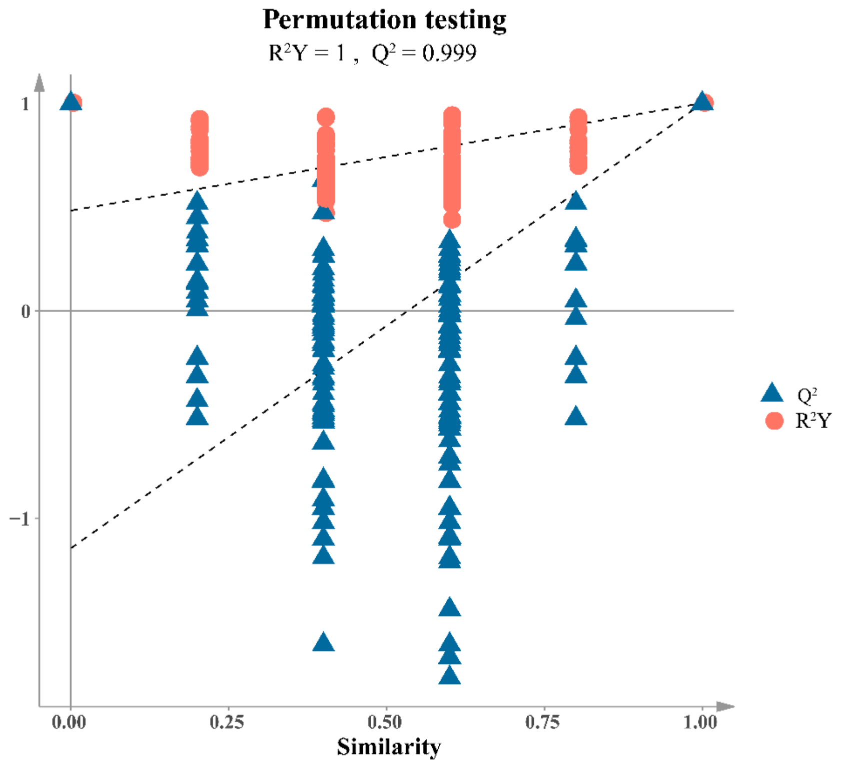

Figure S1 Score plot of OPLS-DA between the BR group and the GBR group and the permutation test charts

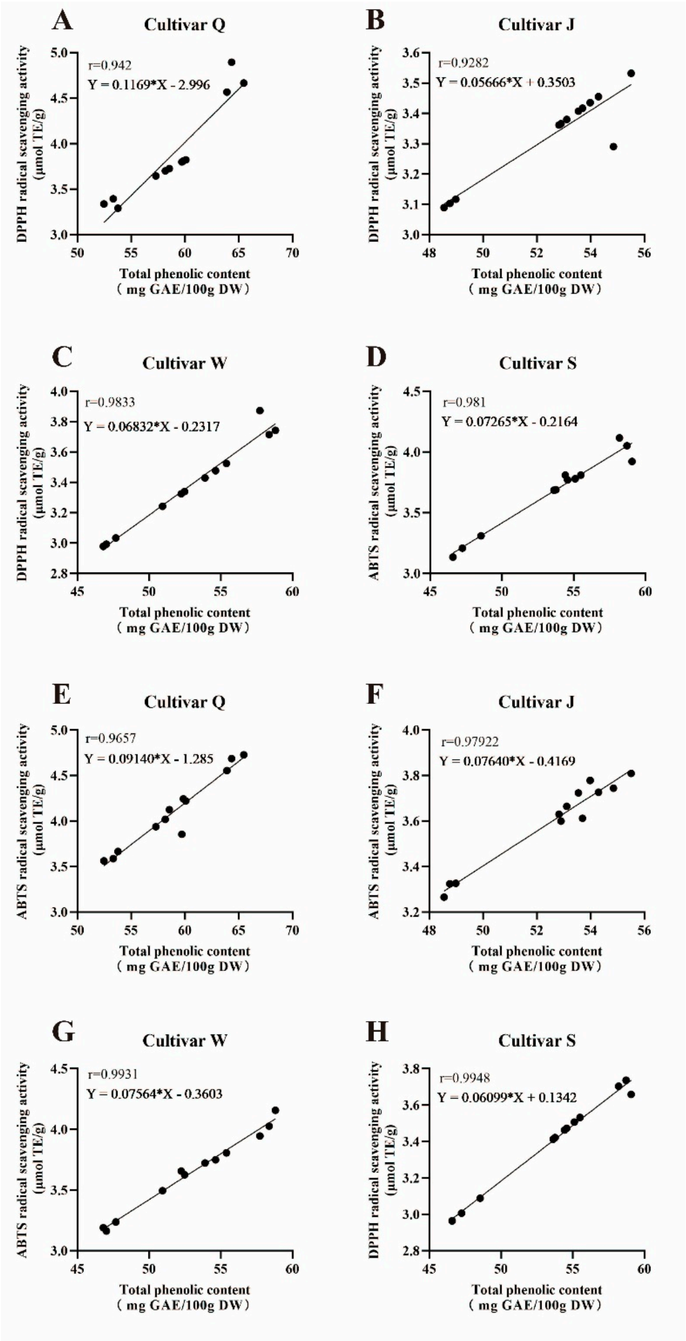

Figure S2 Correlations between total phenolic content and DPPH/ABTS radical scavenging activity in four BR cultivars. Data are presented as mean ± SD (n = 3). All correlations were significant at $p < 0.0001$. (A) Cultivar Q, DPPH radical scavenging activity vs. TPC. (B) Cultivar J, DPPH radical scavenging activity vs. TPC. (C) Cultivar W, DPPH radical scavenging activity vs. TPC. (D) Cultivar S, ABTS radical scavenging activity vs. TPC. (E) Cultivar Q, ABTS radical scavenging activity vs. TPC. (F) Cultivar J, ABTS radical scavenging activity vs. TPC. (G) Cultivar W, ABTS radical scavenging activity vs. TPC. (H) Cultivar S, DPPH radical scavenging activity vs. TPC

Table S5 Germination rates of four rice cultivars at different germination times.

Cultivar	Germination time	Germination rate
	12h	68.12 ± 0.23^{e}
Q	24h	75.25 ± 1.93^{b}
	36h	81.75 ± 2.31^{a}
	12h	57.75 ± 2.28^{g}
J	24h	68.48 ± 1.31^{de}
	36h	71.36 ± 1.16^{cd}
	12h	54.22 ± 1.57^{h}
W	24h	64.33 ± 1.78^{f}
	36h	69.48 ± 2.87^{de}
	12h	62.15 ± 0.26^{f}
S	24h	73.42 ± 1.04^{bc}
	36h	75.28 ± 1.08^{b}

Data are presented as mean ± SD (n = 3). Different lowercase letters in the same column indicate significant differences ($p < 0.05$).

Table S6 the top 20 metabolites based on VIP values

No.	Compound	Molecular Formula	VIP	p Value	rs	FC	m/z	Fragment ions (m/z)	Retention Time (min)	Ion Mode
1	Ferulic acid	$C_{10}H_{10}O_4$	2.18	0.00	0.842**	3.30	193.05	179.0146,134.0337	5.15	$[M-H]^-$
2	Cinnamic acid	$C_9H_8O_2$	2.04	0.00	0.697*	3.6	147.05	147.0456,103.0529	5.07	$[M-H]^-$
3	p-coumaric acid	$C_9H_8O_3$	1.87	0.00	0.863**	2.16	163.04	163.0404,119.0461	4.89	$[M-H]^-$
4	3-Feruloylquinic acid	$C_{17}H_{20}O_9$	1.75	0.00	0.661*	4.50	367.10	193.0516,134.0376	4.09	$[M-H]^-$
5	Alpha-Linolenic acid	$C_{18}H_{30}O_2$	1.70	0.00	-0.845**	0.14	277.22	277.2173,260.2236	12.98	$[M-H]^-$
6	L-(+)-Lysine	$C_6H_{14}N_2O_2$	1.66	0.00	0.855**	2.31	145.10	113.0319,145.0985	0.82	$[M-H]^-$
7	Malonic acid	$C_3H_4O_4$	1.61	0.00	0.794**	3.33	103.00	103.0040,75.0061,	0.96	$[M-H]^-$
8	DL-Histidine	$C_6H_9N_3O_2$	1.54	0.00	0.718*	2.71	154.06	154.0619,93.0439	0.83	$[M-H]^-$
9	L-(+)-glutamine	$C_5H_{10}N_2O_3$	1.43	0.00	0.842**	2.4	145.06	145.0621,127.0513	0.88	$[M-H]^-$
10	L-(+)-Arginine	$C_6H_{14}N_4O_2$	1.39	0.00	0.77**	4.07	173.10	173.1041,131.0826	0.84	$[M-H]^-$
11	Citric acid	$C_6H_8O_7$	1.35	0.00	0.77**	4.73	191.02	111.0042,85.0288	1.90	$[M-H]^-$
12	Succinic acid	$C_4H_6O_4$	1.33	0.00	0.673*	3.18	117.02	117.0194,73.0295	2.24	$[M-H]^-$
13	L-Tryptophan	$C_{11}H_{12}N_2O_2$	1.28	0.00	0.758*	2.39	203.08	203.0827,116.0509	3.49	$[M-H]^-$
14	L-(+)-Glutamic acid	$C_5H_9NO_4$	1.26	0.00	0.673*	2.98	146.05	128.0276,102.0511	0.89	$[M-H]^-$
15	L-Serine	$C_3H_7NO_3$	1.21	0.00	0.855**	2.47	104.04	104.0354,74.0239	0.85	$[M-H]^-$

Table S6 the top 20 metabolites based on VIP values

16	gamma-Aminobutyric acid	$C_4H_9NO_2$	1.18	0.00	0.818**	6.84	102.06	102.0574	0.91	$[M-H]^-$
17	(+)-D-malic acid	$C_4H_6O_5$	1.12	0.00	0.736*	3.90	133.01	133.0139,115.0037	1.21	$[M-H]^-$
18	L-(+)-Aspartic acid	$C_4H_7NO_4$	1.06	0.00	0.636*	2.13	132.03	132.0301,88.0404	0.87	$[M-H]^-$
19	Adenosine	$C_{10}H_{13}N_5O_4$	1.04	0.00	0.709*	4.37	266.09	266.0807,134.0428	2.43	$[M-H]^-$
20	Maltose	$C_{12}H_{22}O_{11}$	1.04	0.00	0.855**	3.06	341.11	161.0451,179.0557	1.16	$[M-H]^-$

Note: All p-values of the differential metabolites were less than 0.001, not equal to 0.

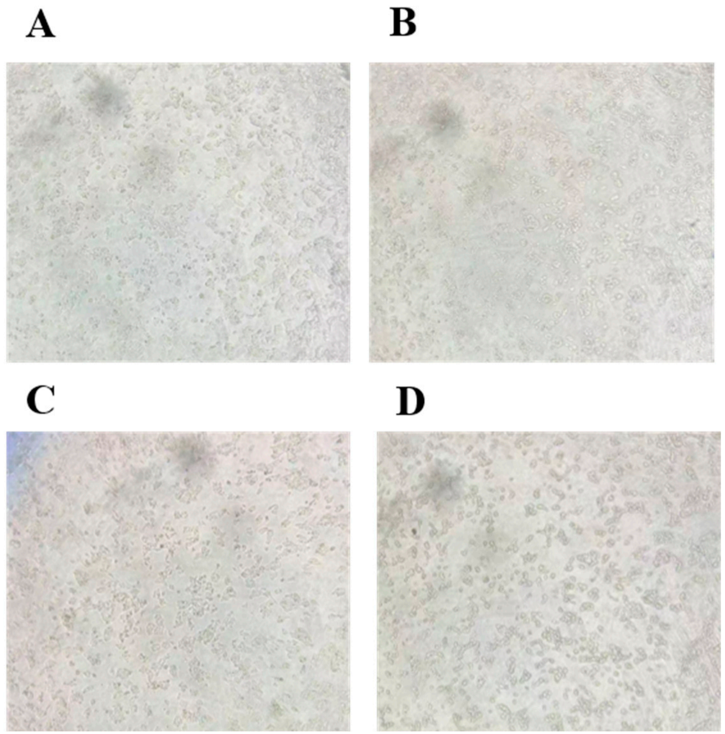

Figure S3 Morphological observations of cholesterol-induced HepG2 cells after 24 h treatment with different concentrations of *p*-coumaric acid. (A) 10 μM; (B) 20 μM; (C) 40 μM; (D) 80 μM.
